# UGCG modulates heart hypertrophy through B4GalT5-mediated mitochondrial oxidative stress and the ERK signaling pathway

**DOI:** 10.1186/s11658-023-00484-3

**Published:** 2023-09-01

**Authors:** Shengyu Cui, Xutao Zhang, Yuhua Li, Shan Hu, Bing Wu, Zhao Fang, Jixian Gao, Ming Li, Haoliang Wu, Bo Tao, Hao Xia, Lin Xu

**Affiliations:** 1 Department of Cardiology, Renmin Hospital of Wuhan University, Wuhan, 430060 China; 2 Cardiovascular Research Institute, Wuhan University, Wuhan, 430060 China; 3 Hubei Key Laboratory of Cardiology, Wuhan, 430060 China; 4 Intensive Care Unit, Wuhan Children’s Hospital (Wuhan Maternal and Child Healthcare Hospital), Tongji Medical College, Huazhong University of Science & Technology, Wuhan, China; 5 Department of Geriatrics, Renmin Hospital of Wuhan University, Wuhan, 430060 Hubei China

**Keywords:** Heart hypertrophy, UGCG, B4GalT5, ERK signaling

## Abstract

Mechanical pressure overload and other stimuli often contribute to heart hypertrophy, a significant factor in the induction of heart failure. The UDP-glucose ceramide glycosyltransferase (UGCG) enzyme plays a crucial role in the metabolism of sphingolipids through the production of glucosylceramide. However, its role in heart hypertrophy remains unknown. In this study, UGCG was induced in response to pressure overload in vivo and phenylephrine stimulation in vitro. Additionally, UGCG downregulation ameliorated cardiomyocyte hypertrophy, improved cardiomyocyte mitochondrial oxidative stress, and reduced the ERK signaling pathway. Conversely, UGCG overexpression in cardiomyocytes promoted heart hypertrophy development, aggravated mitochondrial oxidative stress, and stimulated ERK signaling. Furthermore, the interaction between beta-1,4-galactosyltransferase 5 (B4GalT5), which catalyses the synthesis of lactosylceramide, and UGCG was identified, which also functions as a synergistic molecule of UGCG. Notably, limiting the expression of B4GalT5 impaired the capacity of UGCG to promote myocardial hypertrophy, suggesting that B4GalT5 acts as an intermediary for UGCG. Overall, this study highlights the potential of UGCG as a modulator of heart hypertrophy, rendering it a potential target for combating heart hypertrophy.

## Introduction

The outcome of many heart disorders is heart failure. Heart failure remains a prevalent and life-threatening condition, despite significant advancements in its treatment. Heart hypertrophy, an adaptive response of the myocardium to increased workload caused by various factors, is often considered an early indicator of clinical heart failure [1, 2]. Left ventricular hypertrophy is primarily attributed to the enlargement of cardiomyocytes, leading to enhanced protein synthesis and fetal gene expression, such as atrial natriuretic peptide (ANP), brain natriuretic peptide (BNP), and beta-myosin heavy chain (β-MHC) [3]. Cardiovascular compensatory hypertrophy has been reported to preserve cardiac function in the early stages of heart hypertrophy but prolonged exposure to risk factors ultimately results in decompensation and progression to severe heart failure [4]. Therefore, further study on heart hypertrophy prevention and treatment is crucial.

Glycosylation, a common posttranslational protein modification, can significantly alter the biological characteristics and functions of protein [5]. Numerous biological processes, including as cell-to-cell communication, protein targeting and folding, viral or bacterial infection, cancer, and aging, are significantly influenced by glycosylation [6]. Moreover, abnormal glycosylation of cell surface receptors leads to aberrant myocardial metabolism, contributing to the onset and progression of several heart diseases [7, 8]. UDP-glucose ceramide glycosyltransferase (UGCG), which transfers UDP-glucose to ceramide, is currently the sole enzyme responsible for the de novo production of glucosylceramide (GlcCer) [9]. A recent study [10] reported that UGCG plays a vital role in maintaining beta-adrenergic signaling and contractile capacity in cardiomyocytes. Nevertheless, studies on the role of UGCG in heart disease is limited. The current study focuses on investigating the potential impacts and workings of UGCG in pathologically loaded hearts.

The beta-1,4-galactosyltransferase (B4GalT) gene family, which comprises seven members, is involved in the production of various glycoconjugates and saccharide structures, exhibiting various substrate affinities and end products [11]. B4GalT enzymes, which are type II membrane-bound glycoproteins that are majorly present on the cell surface and in the Golgi apparatus, catalyze the transfer of UDP-galactose in a β1,4 linkage to acceptor sugars [11, 12]. Among the B4GalT genes, B4GalT5 catalyzes the conversion of galactose from UDP-galactose to GlcCer, resulting in lactosylceramide formation [13]. Additionally, B4GalT5, a downstream effector of the UGCG cascade, plays a role in the regulation of UGCG in the heart.

Currently, studies on the role of the UGCG–B4GalT5 axis in pathological heart hypertrophy are limited. Although certain articles have explored the potential effects of downstream products of UGCG on the heart, the specific mechanism of UGCG remains unclear. Moreover, it is yet unknown whether B4GalT5, a key downstream molecule of UGCG, acts as a mediator or collaborator in the effect of UGCG on the heart. Therefore, this study aims to elucidate the mechanisms underlying the role of UGCG in pathological myocardial hypertrophy and investigate the potential joint mediation of UGCG and B4GalT5 in the regulation of cardiac hypertrophy.

## Material and methods

### Animals

Animal experiments were performed in adherence to the National Institutes of Health (NIH) Guide for the Care and Use of Laboratory Animals (revised 1996) and the regulations of Renmin Hospital of Wuhan Hospital (ethical number: 20211203C). Male C57BL/6 mice (8–10 weeks old) were obtained from the Chinese Academy of Medical Sciences’ Institute of Laboratory Animal Science (Beijing, China). All animals were housed in a pathogen-free environment (20–22 °C; 50–5% relative humidity) with unrestricted access to food and water.

Transverse aortic constriction (TAC) surgery was performed to induce pressure-overload-induced hypertrophy and thus create a mouse model of heart hypertrophy. The animals were weighed and divided into groups at random. Based on a previous study, TAC surgery was performed [14]. Briefly, a 7–0 nylon suture was used to ligate the aorta together with a blunted 27-gauge needle, which was removed later.

To elucidate the role of UGCG and B4GalT5 in vivo, mice were exposed to a single intravenous injection of adeno-associated virus (AAV)9 carrying a small hairpin RNA against UGCG (AAV-Sh-UGCG), coding sequence of UGCG (AAV-OV-UGCG) or small hairpin RNA against B4GalT5 (AAV-Sh-B4GalT5), or their corresponding negative control (AAV-Scramble, AAV-Scramble1 and AAV-Scramble2) 3 weeks before the TAC surgery.

### Materials

Antibodies for GAPDH (#5174, 1:1000), ERK (#4695, 1:1000), phosphorylated-ERK1/2 Thr202/Thr204 (#4370, 1:1000), P38 (#9212, 1:1000), and phosphorylated-P38 Thr180/Thr182 (#4511, 1:1000) were obtained from Cell Signaling Technology (Danvers, MA, USA). Anti-collagen I (Col1) (#14,695-1-AP, 1:1000) and anti-UGCG antibodies (#12869-1-AP, 1:500) were obtained from the Proteintech Group (Wuhan, China). Anti-B4GalT5 (#TD3841, 1:500), anti-HA (#M20003, 1:2000), and anti-Flag (#M20008, 1:2000) were obtained from Abmart Inc. (Shanghai, China). Antibodies for ANP (A1609, 1:1000) and BNP (DF6902, 1:1000) were obtained from Abclonal (Wuhan, China) and Affinity Biosciences (Jiangsu, China), respectively. Phenylephrine (PE) and transforming growth factor-beta (TGF-β) were obtained from Sigma-Aldrich (St. Louis, MO, USA). For mitochondrial reactive oxygen species (ROS), Mito-Tracker solution (Beyotime, China) and MitoSOX solution (Invitrogen, USA) were utilized.

### Evaluation of cardiac function

M-mode echocardiograms were performed using a Vinno 6th ultrasound Doppler imaging system (VINNO6, Vinno Corporation, China). Mice were anesthetized with 1.5% isoflurane and then subjected to echocardiographic examination. Subsequently, the parameters of cardiac function, namely left ventricle (LV) internal dimension diastole (LVIDd) and left ventricle posterior wall dimension (LVPWd), were collated for analysis.

### Histological analysis

The mouse heart tissue was excised, soaked in a 10% potassium chloride solution, and then fixed in 4% paraformaldehyde for more than 24 h. Several slices (5 μm thickness) of paraffin-embedded hearts were sectioned and stained with Masson stain for subsequent research such as assessing the cross-sectional area of cardiomyocyte and calculating the heart fibrotic rate. Hematoxylin–eosin (HE) staining was used to evaluate the surface area of cardiomyocytes.

### Separation of neonatal rat cardiomyocytes and cardiac fibroblasts

Neonatal rats that were euthanized at 1–3 days of age provided the samples for the neonatal rat cardiomyocytes. A rapid removal of the heart from the rat chest followed by three phosphate-buffered saline rinses were performed at the start of the analysis. LV tissues were then isolated by removing the extra tissue and slicing the samples into smaller pieces, which were then digested over multiple cycles with 0.125% trypsin. Following this, the samples were treated with a mixture of enzymes (0.1% trypsin and 0.8 mg/ml collagenase II). The supernatant obtained during each digestion was collected and centrifuged to separate the cells, which were then resuspended in a medium and grown in a Petri dish. After 70 min of adherence, the cells floating in the medium suspension were identified as cardiomyocytes and replanted in Petri dishes, whereas cells that adhered to the wall were identified as fibroblasts. The cardiomyocytes were grown in Dulbecco’s modified Eagle medium (DMEM; high glucose) containing 10% fetal bovine serum. PE was used to stimulate cardiomyocytes for 24 h at a dose of 50 μmol/L, while TGF-β was used to stimulate fibroblasts for 24 h at a concentration of 10 ng/mL.

To elucidate the role of UGCG and B4GalT5 in vitro, cells were infected with adenoviruses carrying a small hairpin RNA against UGCG (Ad-Sh-UGCG), coding sequence of UGCG (Ad-OV-UGCG) or small hairpin RNA against B4GalT5 (Ad-Sh-B4GalT5), or their corresponding negative control (Ad-Scramble, Ad-Scramble1 and Ad-Scramble2) 24 h before stimulation.

### Immunofluorescence

The expression levels of UGCG and B4GalT5 in heart or neonatal rat cardiomyocytes were evaluated using an immunofluorescence assay. The specific experimental steps are as previously described [15]. The primary antibodies for UGCG (Proteintech, China), B4GalT5 (Abmart, China), and ctnt (Abcam, UK), as well as the secondary antibody and 4′,6-diamidino-2-phenylindole (DAPI) solution were used for staining. The images were acquired using an immunofluorescence microscope system (NIKON, Japan).

### Immunohistochemistry

The slices were treated with Citrate Antigen Retrieval Solution (PH 6.0) to retrieve the antigen in microwave, placed in the solution of 3% hydrogen peroxide to block endogenous peroxidase, and then incubated with 3% bovine serum albumin for 30 min and with anti-α-SMA antibody (Cell Signaling Technology, USA) overnight. Finally, the slices were incubated with horseradish peroxidase (HRP)-conjugated secondary antibody for 60 min at room temperature, and with enough diaminobenzidine for 2–5 min at room temperature until a brown color developed.

### Measurement of ROS production

For the measurement of mitochondrial ROS, cells were incubated with a 5 mM MitoSOX solution for 15 min, and images were detected using an immunofluorescence microscope system (NIKON, Japan).

### Transmission electron microscope (TEM) detection

The cells were post-fixed with 1% buffered osmium tetroxide for 2 h at 4 °C after being prefixed in 2.5% glutaraldehyde in PBS overnight. After two final 15 min rinses in 100% ethanol, the specimens were dehydrated using graded ethanol. The specimens were collected for ultrathin slices after being embedded, and they underwent double staining with uranyl acetate and lead citrate. A transmission electron microscope (FEI, TECNAI G2 20 TWIN, USA) was used to examine slices.

### Measurement of mitochondrial membrane potential (MMP)

Cellular MMP was discovered using the JC-1 reagent. The cells were reacted with JC-1 solution at 37 °C for 30 min, per the manufacturer’s instructions, and DAPI solution (Sigma, USA) was used to color the nucleus. Lastly, a fluorescence microscope was used to observe and record changes in JC-1 fluorescence. When the MMP dropped, JC-1 aggregates (red fluorescence) transformed into JC-1 monomers (green fluorescence).

### Quantitative real-time PCR (qRT–PCR)

For total RNA analysis, TaKaRa (JapanRNAiso)’s Plus was employed. Using the Servicebio RT First Strand cDNA Synthesis Kit (Servicebio, China) in accordance with the manufacturer’s instructions, complementary DNA (cDNA) was generated. An ABI ViiA7 Real-Time PCR machine and Servicebio 2×SYBR Green qPCR Master Mix kit (low ROX) were used to perform qRT–PCR (Applied Biosystems, USA). The primers used for qRT–PCR are listed below:

**Table:** 

ANP	5′-ACCTGCTAGACCACCTGGAG-3′	5′-CCTTGGCTGTTATCTTCGGTACCGG-3′
BNP	5′-GAGGTCACTCCTATCCTCTGG-3′	5′-GCCATTTCCTCCGACTTTTCTC-3′
β-MHC	5′-CCGAGTCCCAGGTCAACAA-3′	5′-CTTCACGGGCACCCTTGGA-3′

### Western blot assays

Western blot assays were performed following a previous report [16]. Briefly, sodium dodecyl sulfate–polyacrylamide gel electrophoresis (SDS–PAGE) was used to separate the proteins obtained from the hearts and cells before they were transferred to polyvinylidene difluoride (PVDF) membranes. The membranes were then blocked with protein-free fast-blocking solution (Epizyme Biotech, China) and incubated with primary antibodies overnight. Following this, the membranes were then incubated with secondary antibodies conjugated with HRP for 90 min at room temperature. Finally, using a ChemiDoc XRS + system, the improved chemiluminescence reagent was employed to visualize the membranes (BIO-RAD, USA). Additionally, Image J was used to quantify protein bands.

### Statistical analysis

The GraphPad Prism software (Version 9.0) was used to analyze the data. The data are presented as mean ± standard deviation. To compare multiple groups, one-way analysis of variance (ANOVA) was used, followed by the Tukey’s post hoc test. For comparison between two groups, an unpaired Student’s *t*-test was performed. *p*-values < 0.05 were used as the threshold for statistical significance.

## Results

### UGCG was upregulated in the pressure overloaded heart

**Fig. 1:**
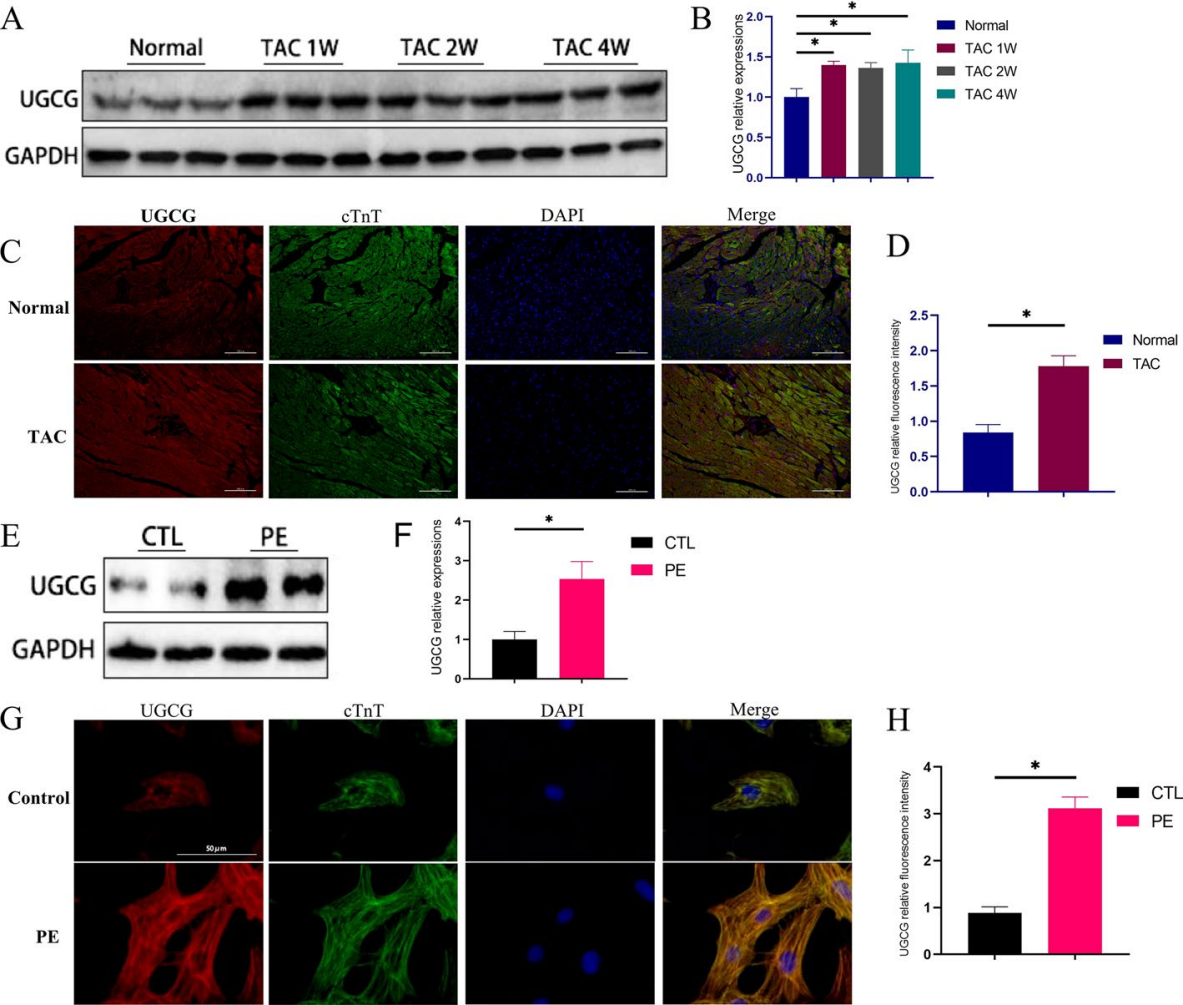
UGCG expression is increased in the pressure overload heart. **A**, **B** Western blot assays showed the expression level of UGCG at different time points and corresponding statistical analysis histogram. **C**, **D** Immunofluorescence staining showed the expression of UGCG in different heart groups and corresponding statistical analysis diagrams. **E**, **F** Western blot assays showed the expression level of UGCG in primary cardiomyocytes in vitro and corresponding statistical analysis histogram. **G**, **H** Immunofluorescence staining showed the expression of UGCG in primary cardiomyocytes in vitro and corresponding statistical analysis histogram. *N* = 6, *p < 0.05. UGCG: UDP-glucose ceramide glycosyltransferase; TAC: Transverse aortic constriction; PE: Phenylephrine

First, we detected the expression levels of UGCG in the hearts of pressure overloaded mice and normal mice. Western blot revealed increased UGCG levels in the hearts of mice after TAC surgery (Fig. 1A, B). Additionally, immunofluorescence also detected higher levels of UGCG expression in hypertrophic hearts (Fig. 1C, D). Furthermore, UGCG was identified in primary cardiomyocytes in vitro. Western blot (Fig. 1E, F) and immunofluorescence (Fig. 1G, H) also revealed that UGCG levels in PE-stimulated cardiomyocytes were significantly higher than in the control group.

### Downregulation of UGCG ameliorated heart hypertrophy in vivo

**Fig. 2:**
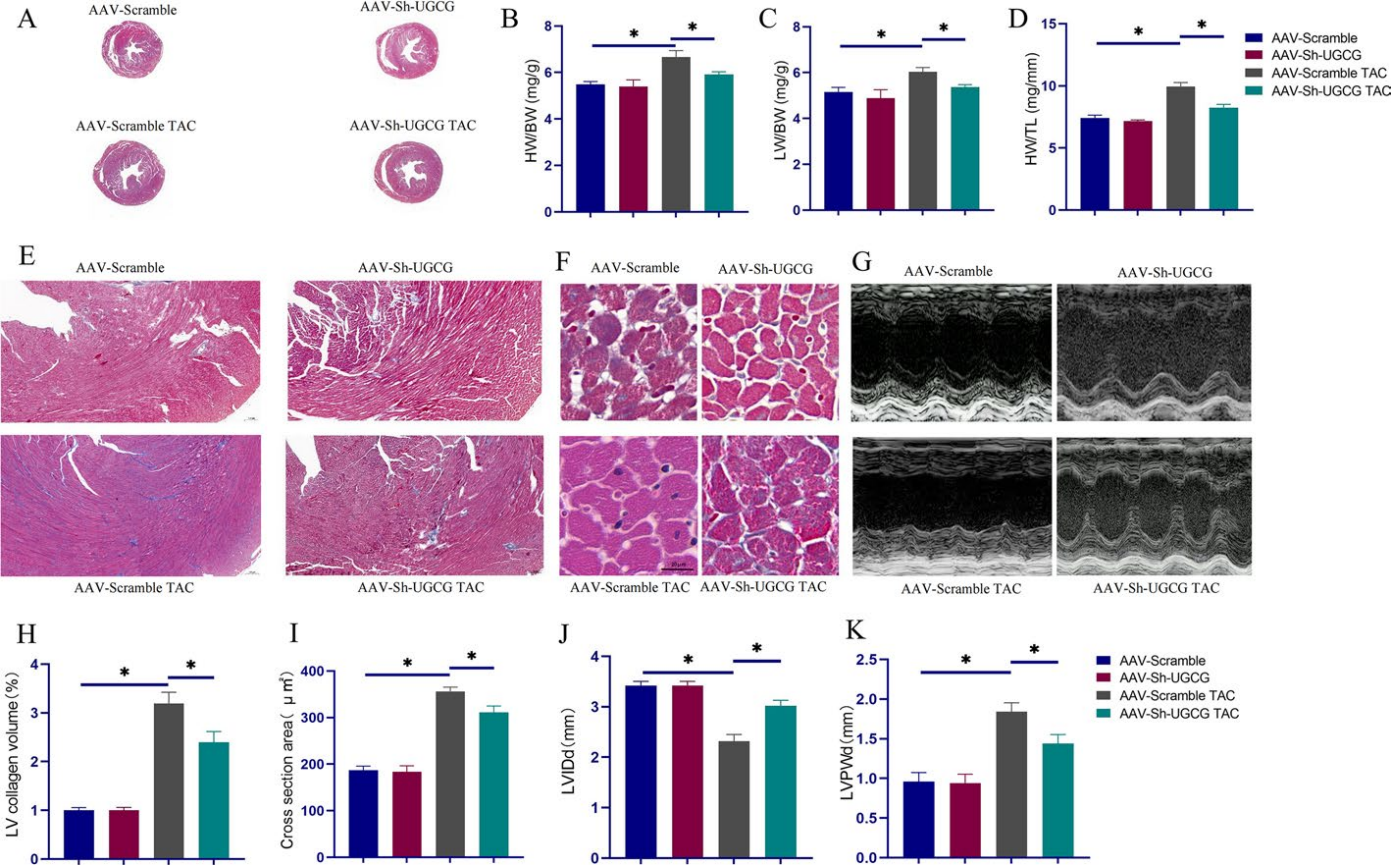
Inhibition of UGCG expression in the heart relieved pathological remodeling of myocardial hypertrophy. **A** Masson staining showed the overall degree of myocardial hypertrophy and fibrosis. **B**–**D** Statistical charts of HW/BW, LW/BW, and HW/TL in different groups. **E**, **H** Masson staining showed fibrosis at the same site in the hearts of different groups of mice and corresponding statistical analysis histogram. **F**, **I** The representative pictures of the size of the cross-sectional area of cardiomyocytes in the heart and corresponding statistical analysis histogram. **G**, **J**, **K** Echocardiogram and index results of each group. *N* = 5, *p < 0.05. AAV-Scramble: negative control of AAV-Sh-UGCG; AAV: Adeno-associated virus; Sh: Small hairpin; LVIDd: Left ventricle internal dimension diastole; LVPWd: Left ventricle posterior wall dimension

To investigate UGCG’s potential contribution to pathological heart hypertrophy, we developed adeno-associated viruses that inhibited UGCG expression. Notably, the inhibition of UGCG expression in the heart significantly reduced myocardial hypertrophy induced by pressure overload, which was further validated by the improved ventricular wall hypertrophy (Fig. 2A), lower heart weight to body weight ratios (HW/BW), decreased left ventricular weight to body weight ratios (LW/BW), and decreased heart weight to tibial length ratios (HW/TL) (Fig. 2D). Additionally, UGCG inhibition mitigated additional hypertrophic alterations, including the degree of cardiac fibrosis (Fig. 2E, H), the cross-sectional size of myocardial cells (Fig. 2I), and echocardiograms (Fig. 2J, K). Overall, these findings suggest that UGCG inhibition alleviated the pathogenic alterations associated with heart hypertrophy.

**Fig. 3:**
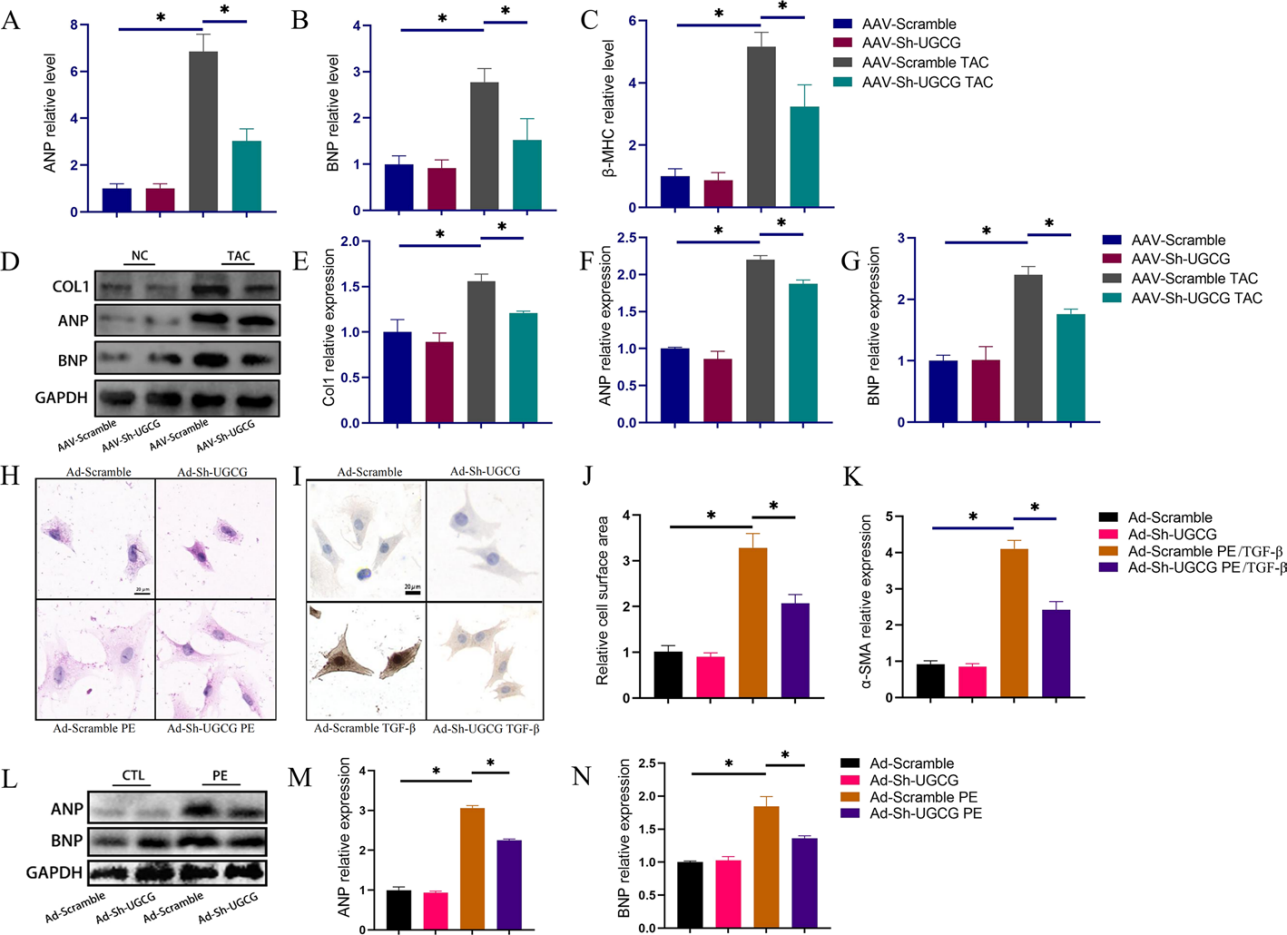
Inhibition of UGCG expression ameliorated heart hypertrophy and heart fibrosis. **A**–**C** mRNA levels of ANP, BNP, and β-MHC in hearts from different groups (*N* = 6). **D**–**G** Western blot results of Col1, ANP, BNP expression levels in hearts from different groups (*N* = 3). **H**, **J** HE staining showed the cell surface area of cardiomyocytes from different groups (*N* = 6). **I**, **K** Immunohistochemical images of α-SMA staining in heart fibroblasts from different groups (*N* = 6). **L**, **N** Western blot results of ANP and BNP expression levels in cardiomyocytes from different groups (*N* = 3). *p < 0.05. AAV-Scramble: Negative control of AAV-Sh-UGCG; Ad-Scramble: Negative control of Ad-Sh-UGCG; ANP: Atrial natriuretic peptide; BNP: Brain natriuretic peptide; β-MHC: β-myosin heavy chain; Col1: Collagen I; Ad: Adenovirus; TGF-β: Transforming growth factor-beta

We also examined changes in the molecular biology of the hearts of each group. ANP, BNP, and β-MHC mRNA levels were increased owing to the pressure overload but were decreased on UGCG inhibition (Figs. 3A–C). Additionally, western blot revealed a beneficial effect of UGCG inhibition, which was evidence by the drop in Col1, ANP, and BNP levels in the AAV-Sh-UGCG TAC group compared with the AAV-Scramble TAC group (Fig. 3D–G).

### Downregulation of UGCG ameliorated cardiomyocyte hypertrophy in vitro

We isolated primary cells and conducted in vitro tests to further investigate the impact of UGCG on cardiomyocyte hypertrophy. PE stimulation significantly expanded the surface area of cardiomyocytes, as seen in Fig. 3H, J, whereas the surface area size of cardiomyocytes was improved after UGCG inhibition. It is also worth noting that UGCG inhibition significantly reduced fibroblast trans-differentiation (which is positively correlated with α-SMA expression [17]) induced by TGF-β (Fig. 3I, K). This finding provides a partial insight into the mechanism by which UGCG inhibition impacts cardiac fibrosis. Furthermore, the downregulation of UGCG in cardiomyocytes stimulated with PE led to decreased levels of ANP and BNP, suggesting that UGCG suppression may attenuate heart hypertrophy (Fig. 3L–N).

### Upregulation of UGCG exacerbated heart hypertrophy

**Fig. 4:**
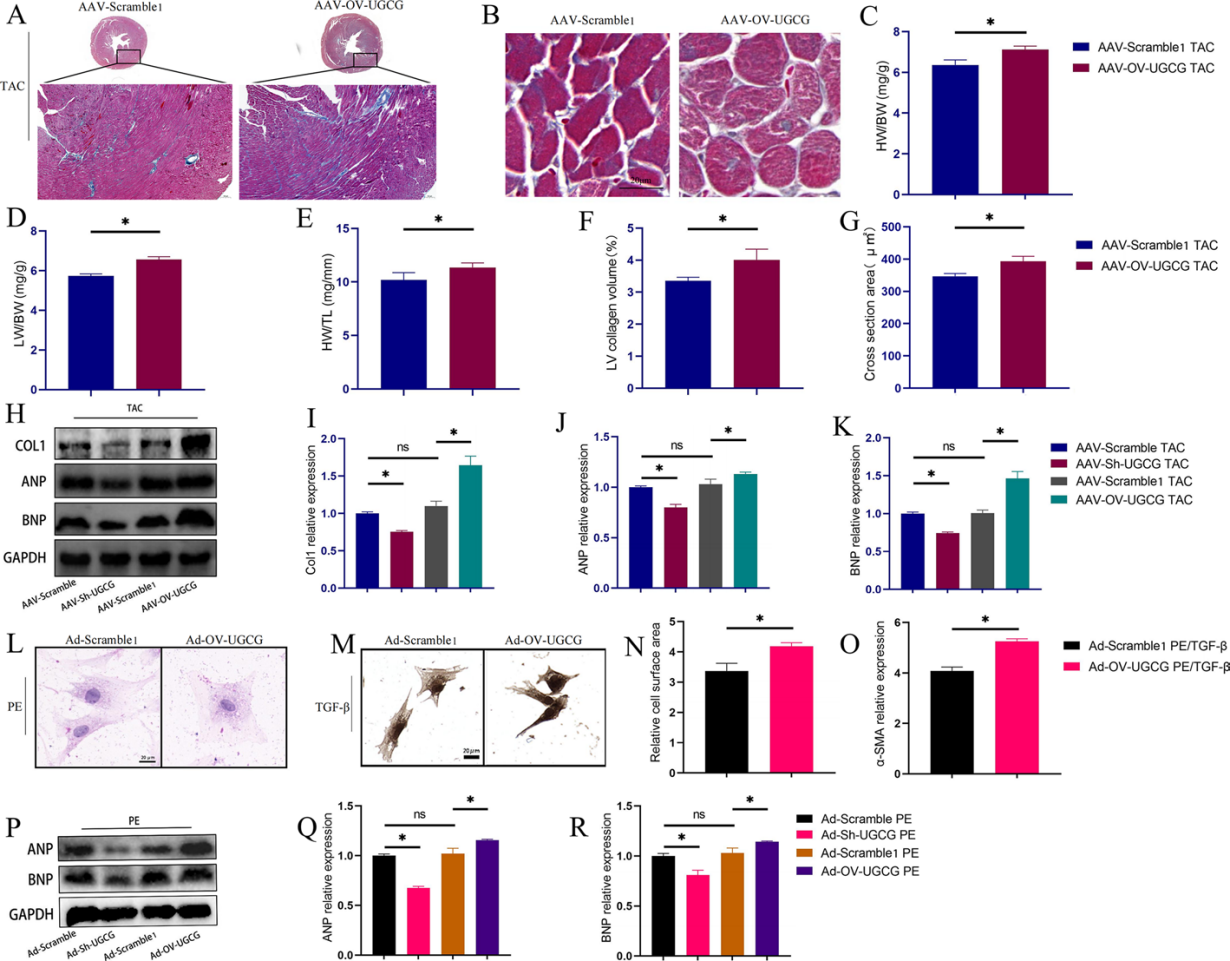
Overexpression of UGCG aggravated heart remodeling. **A**, **F** Masson staining showed the overall degree of myocardial hypertrophy and fibrosis (*N* = 5). **B**, **G** The representative pictures of the size of the cross-sectional area of cardiomyocytes in the heart and corresponding statistical analysis histogram (*N* = 5). **C**–**E** Statistical charts of HW/BW, LW/BW, and HW/TL in different groups (*N* = 5). **H**–**K** Western blot results of Col1, ANP, and BNP expression levels in hearts from different groups (*N* = 3). **L**, **N** HE staining showed the cell surface area of cardiomyocytes from different groups (*N* = 6). **M**, **O** Immunohistochemical images of α-SMA staining in heart fibroblasts from different groups (*N* = 6). **P**–**R** Western blot results of ANP, BNP expression levels in cardiomyocytes from different groups (*N* = 3). *p < 0.05, ns: No significance. AAV-Scramble1: Negative control of AAV-OV-UGCG; Ad-Scramble1: Negative control of Ad-OV-UGCG; HW/BW: Heart weight/body weight; LW/BW: Left ventricle weight/body weight; HW/TL: Heart weight/tibia length; LV: Left ventricle

To further unravel the role of UGCG in pathologic heart hypertrophy, we overexpressed UGCG in mouse hearts and observed the pathologic remodeling of hearts after TAC modeling. As shown in Fig. 4, the overexpression of UGCG in the heart exacerbates cardiac hypertrophy (Fig. 4A, C–E), as evidence by an increase in the cross-sectional area of cardiomyocytes (Fig. 4B, G) and rate of fibrosis (Fig. 4A, F). Moreover, the expressions of Col1, ANP, and BNP in the heart were significantly higher in the UGCG overexpression group than that in the negative virus control group (Fig. 4H–K).

In vitro experiments, the overexpression of UGCG not only exacerbated cardiomyocytes hypertrophy but also increased the trans-differentiation of cardiac fibroblasts (Fig. 4L, N). Additionally, the expression of ANP and BNP in cardiomyocytes was also significantly elevated upon UGCG overexpression (Fig. 4P–R).

Taken together, these results suggested that UGCG promotes pathological heart hypertrophy while the inhibition of UGCG attenuates the progression of heart hypertrophy.

### UGCG interacts with B4GalT5

**Fig. 5:**
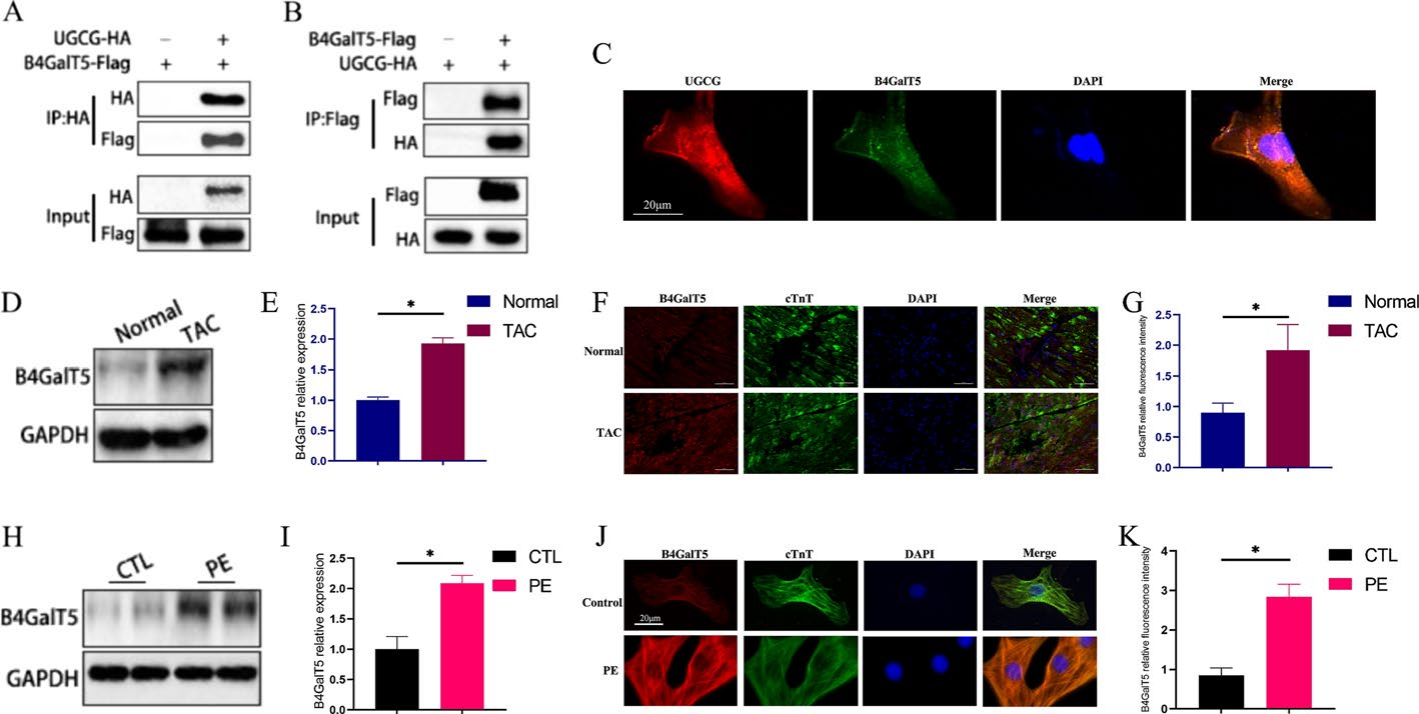
UGCG interacts with B4GalT5. **A**, **B** Representative western blots performed with flag or HA antibody after co-immunoprecipitation (IP) of UGCG (A) or B4GalT5 (**B**) from HEK293t whole-cell lysates using flag antibody or HA antibody, respectively. **C** Representative confocal images of UGCG and B4GalT5 co-localization in primary cardiomyocytes. **D**, **E** Representative western blots and statistical analysis histogram reflecting B4GalT5 expression in hearts (*N* = 3). **F**, **G** Immunofluorescence images of B4GalT5 staining in hearts from different groups (*N* = 5). **H**, **I** Representative western blots and statistical analysis histogram reflecting B4GalT5 expression in cardiomyocytes (*N* = 3). **J**, **K** Immunofluorescence images of B4GalT5 staining in cardiomyocytes from different groups (*N* = 5). *p < 0.05. B4GalT5: Beta-1,4-galactosyltransferase 5

Considering UGCG, the primary rate-limiting enzyme in the glucosylceramide biosynthesis pathway [18], and B4GalT5, which can convert glucosylceramides into potentially inactive lactosylceramides [19], we hypothesized that both enzymes form a complex and play a role in the glycosylation process. Accordingly, immunoprecipitation in HEK293t cells validated the binding of UGCG to B4GalT5 (Fig. 5A, B). We also performed UGCG and B4GalT5 co-staining in primary cardiomyocytes and found that UGCG was co-localized with B4GalT5 (Fig. 5C).

Furthermore, we investigated the expression level of B4GalT5 in pathological myocardial hypertrophy, revealing an elevated B4GalT5 expression in hypertrophied hearts (Fig. 5D–G) and PE-stimulated hypertrophic cardiomyocytes (Fig. 5H–K) compared with the normal group.

### Inhibition of B4GalT5 ameliorated the increased myocardial hypertrophy caused by overexpression of UGCG

**Fig. 6:**
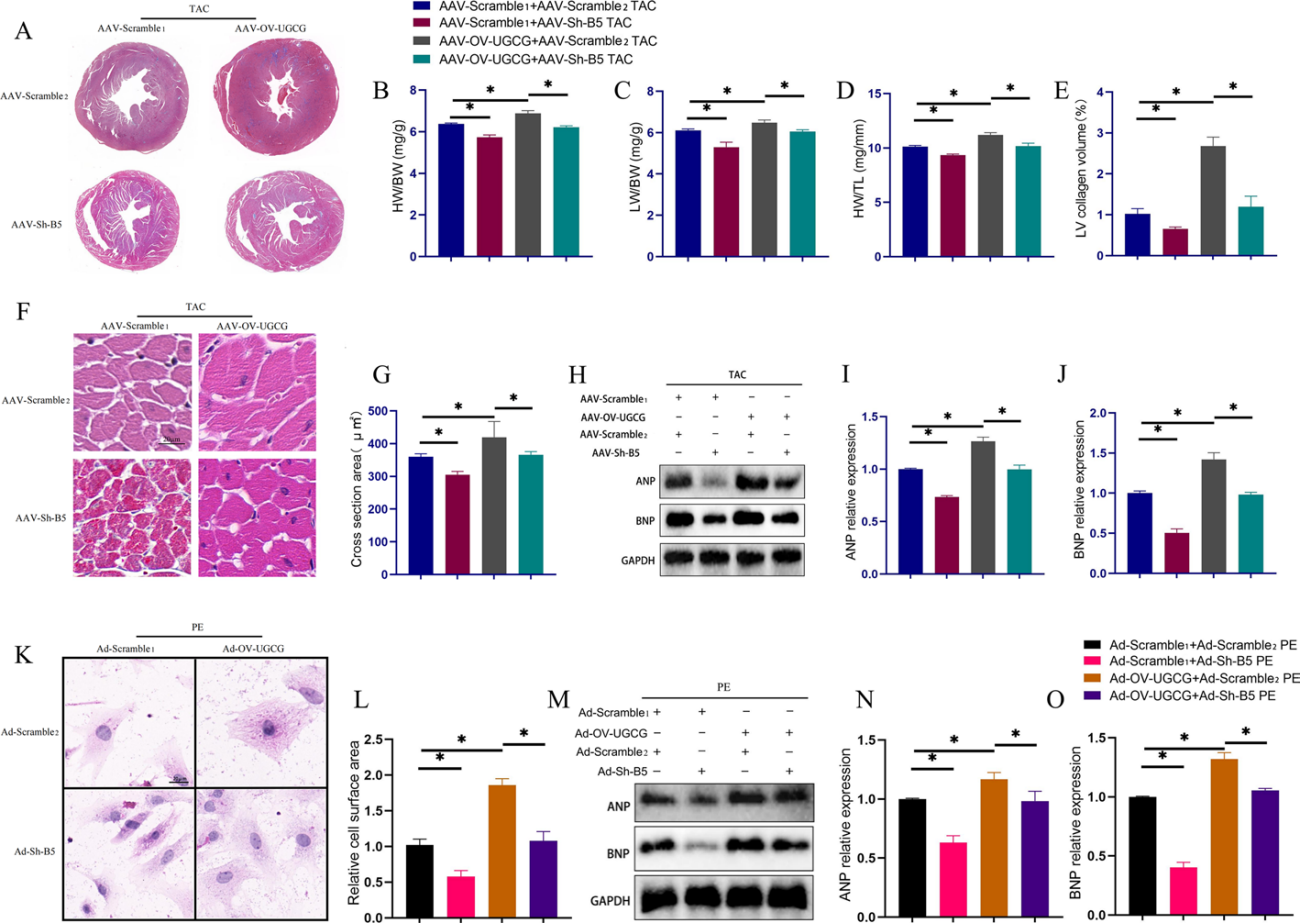
B4GalT5 was involved in the regulation of myocardial hypertrophy by UGCG. **A** Masson staining showed the overall degree of myocardial hypertrophy and fibrosis (*N* = 5). **B**–**E** Statistical charts of HW/BW, LW/BW, HW/TL, and LV collagen volume in different groups (*N* = 5). **F**, **G** The representative pictures of the size of the cross-sectional area of cardiomyocytes in the heart and corresponding statistical analysis histogram (*N* = 5). **H**–**J** Representative western blots and statistical analysis histogram reflecting ANP and BNP expressions in hearts (*N* = 3). **K**, **L** HE staining showed the cell surface area of cardiomyocytes from different groups (*N* = 5). **M**–**O** Western blot results of ANP and BNP expression levels in cardiomyocytes from different groups (*N* = 3). *p < 0.05. AAV-Scramble2: Negative control of AAV-Sh- B4GalT5; Ad-Scramble2: Negative control of Ad-Sh-B4GalT5; B5: B4GalT5

As B4GalT5 can function as a downstream metabolic molecule of UGCG, it is speculated to be involved in the pro-hypertrophic effects of UGCG. To investigate the impact of B4GalT5 inhibition on UGCG’s action, we overexpressed UGCG while simultaneously inhibiting B4GalT5 expression using virally packaged shRNA. As shown in Fig. 6, compared with the UGCG overexpressing group, the group that received a simultaneous administration of UGCG overexpression and B4GalT5 inhibition showed improved myocardial hypertrophy (Fig. 6A–D) and myocardial fibrosis (Fig. 6A, E), along with a decreased myocardial size (Fig. 6F, G) and inhibited expression of ANP and BNP (Fig. 6H–J). Similar effects were observed in cardiomyocytes in vitro (Fig. 6K–O).

### UGCG and B4GalT5 synergistically participated in mitochondrial oxidative stress

**Fig. 7:**
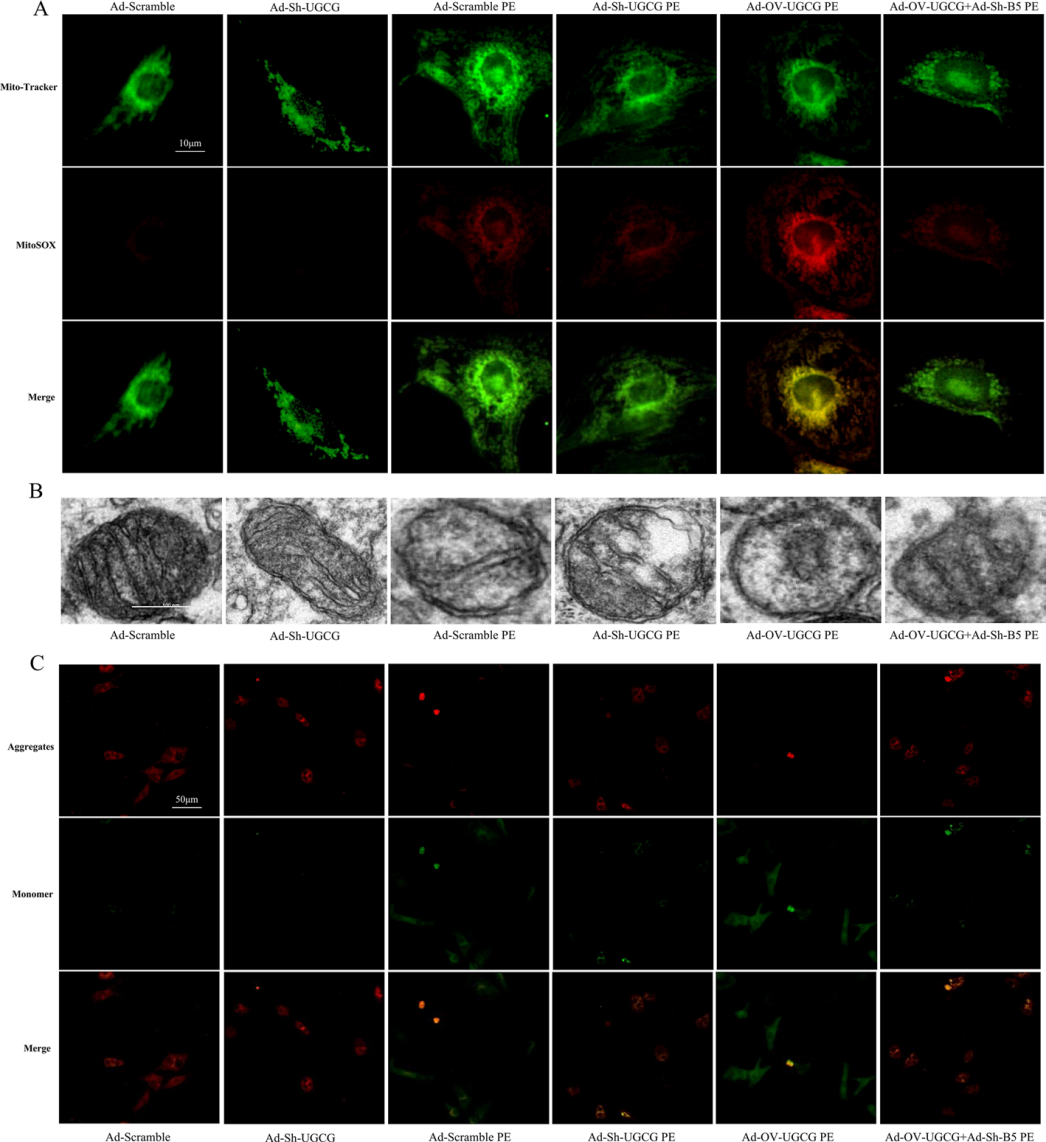
UGCG and B4GalT5 synergistically participated in mitochondrial oxidative stress. **A** Mito-Tracker and MitoSOX double staining to detect the mitochondrial ROS levels in cardiomyocytes in vitro. **B** Transmission electron microscopy showing the mitochondrial morphological changes. **C** Representative images of JC-1 staining. *N* = 5

Recently, it has been reported that UGCG is involved in the regulation of mitochondrial oxidative stress level [20]. Therefore, we speculated that UGCG and B4GalT5 formed a complex to regulate mitochondrial ROS levels and thereby modulate myocardial hypertrophy. As shown in Fig. 7A, the inhibition of UGCG weakened Mito-Tracker and MitoSOX co-localization, indicating mitochondrial ROS levels were reduced after UGCG inhibition, while they were increased after UGCG overexpression. Notably, when B4GalT5 inhibition was applied in conjunction with UGCG overexpression, mitochondrial ROS levels were improved. Furthermore, TEM was used to examine the mitochondrial morphological changes (Fig. 7B): In hypertrophic cardiomyocytes stimulated by PE, mitochondrial cristae were broken and significantly reduced. However, in hypertrophic cardiomyocytes with UGCG inhibition, mitochondrial cristae were partially restored. Moreover, mitochondrial cristae disappeared completely in cardiomyocytes that overexpressed UGCG, while the internal mitochondrial structure showed recovery in cardiomyocytes that simultaneously overexpressed UGCG and suppressed B4GalT5. JC-1 staining was applied for the detection of MMP (Fig. 7C). Silencing UGCG expression or B4GalT5 inhibition, in combination with UGCG overexpression, resulted in increased mitochondrial membrane potential in cardiomyocytes subjected to PE treatment. Conversely, forced UGCG expression had the opposite effect on mitochondrial membrane potential. These results suggested that UGCG and B4GalT5 could synergistically regulate mitochondrial oxidative stress.

### ERK signaling might partially explain the effects of UGCG and B4GalT5

**Fig. 8:**
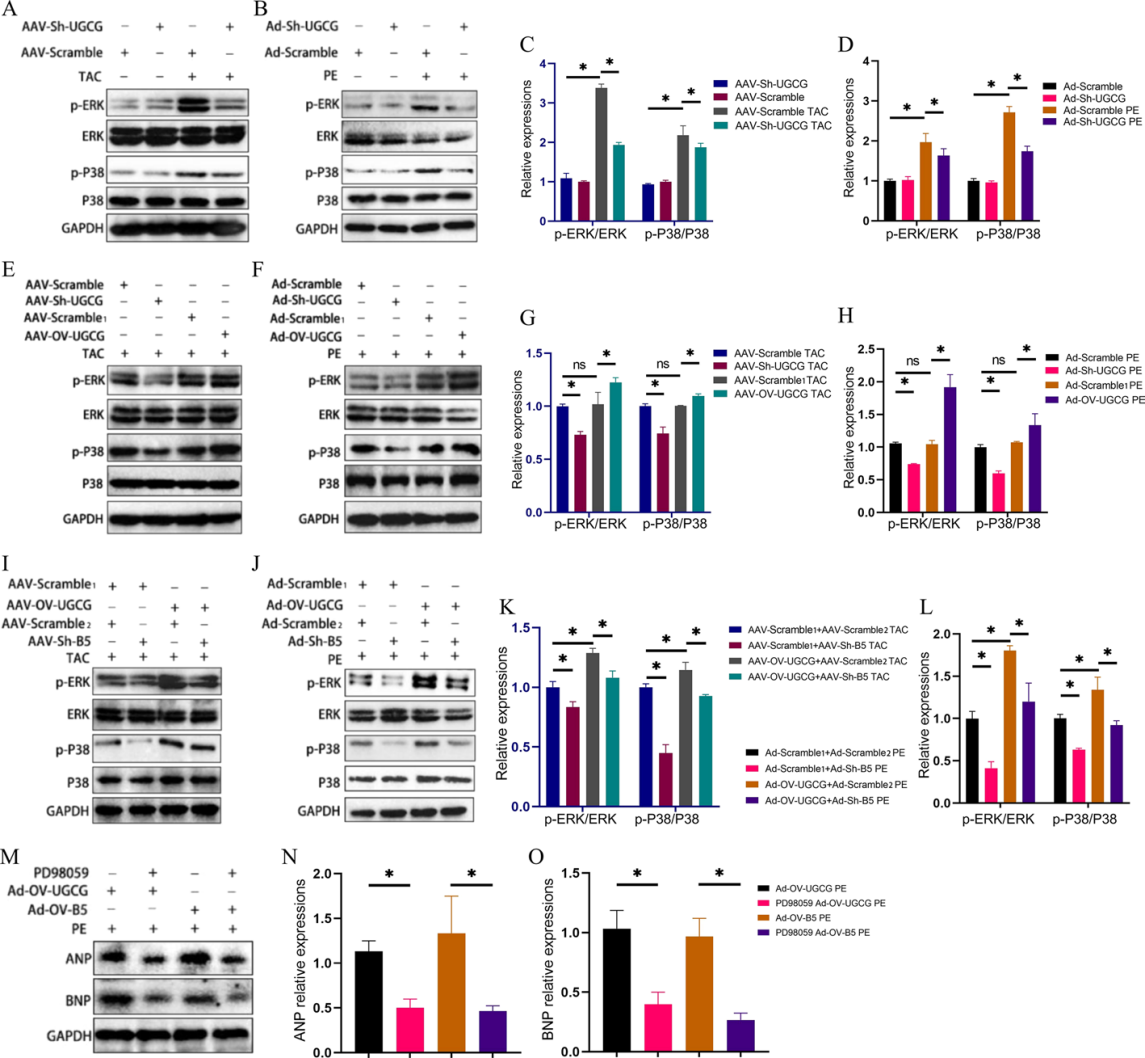
ERK signaling participated in the effects of UGCG and B4GalT5 on heart hypertrophy. **A**, **C** Representative western blots and statistical analysis histogram reflecting ERK signaling in hearts. **B**, **D** Representative western blots and statistical analysis histogram reflecting ERK signaling in cardiomyocytes in vitro. **E**, **G** Representative western blots and statistical analysis histogram reflecting ERK signaling in hearts from different group. **F**, **H** Representative western blots and statistical analysis histogram reflecting ERK signaling in cardiomyocytes in vitro (*N* = 3). **I**, **K** Representative western blots and statistical analysis histogram reflecting ERK signaling in hearts. **J**, **L** Representative western blots and statistical analysis histogram reflecting ERK signaling in cardiomyocytes in vitro. **M**–**O** Western blot assays showed the expressions of ANP and BNP in the presence of PD98059, an ERK signaling inhibitor. *N* = 3, *p < 0.05, ns: No significance; B5: B4GalT5

Considering the close association among ERK signaling, ROS levels [21], and myocardial hypertrophy [22, 23], and the existence of potential links between ERK signaling, UGCG [24, 25], and B4GalT5 [26], we examined the expression of ERK and P38-related molecules. The findings revealed a positive correlation between the status of ERK signaling and UGCG expression (Fig. 8A–H). Additionally, corresponding changes in ERK signaling were observed following the inhibition of B4GalT5 (Fig. 8I–L). Furthermore, the increase in ANP and BNP expression induced by the overexpression of UGCG or B4GalT5 was significantly reduced after the addition of PD98059, an ERK signal inhibitor (Fig. 8M–O). Taken together, these findings strongly suggest that the involvement of UGCG and B4GalT5 in myocardial hypertrophy may be mediated through ERK signaling.

## Discussion

Heart failure is a complex condition, and pathological heart hypertrophy is one of its significant contributors [27]. Recent studies have made remarkable progress in identifying molecular targets and signaling pathways in heart hypertrophy. However, clinical pharmacological approaches for treating heart hypertrophy remain unsatisfactory. In our current study, we demonstrated that UGCG and B4GalT5 were remarkably upregulated in heart hypertrophy brought on by pressure overload in vivo or PE in vitro. Additionally, immunoprecipitation analysis confirmed that B4GalT5 binds to UGCG. Importantly, the inhibition of UGCG ameliorated the pathological changes associated with heart hypertrophy, and blocking B4GalT5 attenuated the hypertrophic effects of UGCG. Furthermore, alterations in ERK signaling were observed along with changes in UGCG and B4GalT5 expression. Together, these findings suggest the crucial roles of UGCG and B4GalT5 in regulating cardiac remodeling, highlighting potential treatment approaches for pathological ventricular hypertrophy.

Currently, the majority of UGCG research is focused on tumors [28–30] and their association with tumor growth and glucose metabolism [31]. Pathological changes, such as unbalanced cell proliferation, survival, and metabolism, occur in the myocardium when heart hypertrophy progresses [32]. To maintain mitochondrial energy metabolism equilibrium during hypertrophy, the expression of genes coding for proteins involved in energetics and oxidative phosphorylation is often dysregulated [33]. Notably, recent studies have linked UGCG to oxidative phosphorylation and cell energy metabolism [20, 29], further emphasizing the need to consider UGCG’s role in myocardial hypertrophy. Our findings support the involvement of UGCG in pathological myocardium hypertrophy, wherein UGCG inhibition reduced the development of myocardial hypertrophy and UGCG overexpression accelerated this condition. Previous studies have also hinted at UGCG’s potential contribution to pathological heart hypertrophy. For instance, a glucosylceramide synthase inhibitor was reported to have a protective effect on pathological heart hypertrophy [34], which is consistent with our findings. Collectively, these findings highlight the negative effects of glucosylceramide in pathological heart hypertrophy. However, investigations have also revealed that animals with a cardiomyocyte-specific UGCG deletion exhibit decreased contractile capacity in response to dobutamine stress in 9–10-week-old mice. Even in the absence of baseline circumstances, older UGCG −/− mice experienced significant cardiac failure and left ventricular enlargement. The crucial function of UGCG in normal physiological settings is supported by the diminished beta-adrenergic stimulation observed in cardiomyocytes from UGCG −/− mice and the reduced uptake and trafficking of β1-adrenergic receptors upon UGCG knockdown [10]. While our results may appear inconsistent with those of Andersson et al. [10], it should be noted that the timing of UGCG regulation differs between our studies. Therefore, by integrating the findings from our experiments with earlier studies, we propose that UGCG is crucial for the growth and development of the heart, but high levels of UGCG expression could have negative effects on the heart when it is stimulated in a pathological way, leading to remodeling. Therefore, theoretically, when the heart is exposed to pathogenic stimulation, UGCG inhibition may result in less cardiac remodeling and good outcomes.

UGCG, the first limiting enzyme in the glucosylceramide biosynthesis pathway [35], and B4GalT5, which can convert glucosylceramides into potentially inactive lactosylceramides [13], interact to form a complex. The interaction between the molecules of UGCG and B4GalT5, which are on the same metabolic pathway, has not been thoroughly studied. Our findings revealed a collaborative relationship between the two molecules, with B4GalT5 acting as a mediator for UGCG to promote myocardial hypertrophy, adding complexity to their biological roles. As B4Galt5 has the potential to promote tumor growth, tumor illnesses are the main focus of current research [11, 12]. Studies on the role of lactosylceramide in the heart [36, 37] suggest that B4Galt5 may play a significant role in heart disease. Lactosylceramide is a downstream metabolite of B4GalT5. Novgorodov et al. [37] reported that lactosylceramide could efficiently inhibit mitochondrial activity in the study of diabetic cardiomyopathy, indicating that lactosylceramide is the primary sphingolipid causing mitochondrial abnormalities in diabetic hearts. Moreover, Mishra et al. [36] further reported the detrimental effects of lactosylceramide on myocardia, particularly its promotion of heart hypertrophy and reactive oxygen production. Notably, mitochondrial damage is important for inducing intracellular oxidative stress response [38]. Consistent with previous reports, this study also reports that the inhibition of UGCG–B4GalT5 axis can improve mitochondrial damage, restore mitochondrial membrane potential, and reduce cardiomyocyte hypertrophy. Therefore, the regulatory effect of B4GalT5 on mitochondrial homeostasis and cardiomyocytes hypertrophy can be reaffirmed.

The ERK signaling pathway is a well-known signal transduction system involved in heart hypertrophy, influencing cell proliferation [39], differentiation [40], apoptosis [41], oxidative stress [42], immunological inflammation [43], and other pathological processes. Moreover, ERK signaling pathway has been reported to promote heart hypertrophy and heart failure [44, 45]. Additionally, ERK signals play a role in the function of UGCG, and B4GalT5 has a role in ERK-related signals. For instance, the inhibition of the glucosylceramide synthase suppresses cisplatin-induced cholangiocarcinoma apoptosis via the inhibition of the ERK signaling pathway [25] while downregulation of B4GalT5 partially inhibits M1 macrophage-mediated adipose tissue inflammation by suppressing ERK signaling [46]. Thus, the UGCG–B4GalT5 axis appears to promote the ERK signaling, and our results also confirm that the activation of the UGCG–B4GalT5 axis is accompanied by the activation of ERK, therefore promoting the progression of myocardial hypertrophy. Moreover, downregulating UGCG leads to mitigated ERK activation, accompanied by reduced myocardial hypertrophy and heart remodeling. Consequently, our findings suggest that the UGCG and B4GalT5 molecular axes may control ERK signaling, ultimately influencing myocardial hypertrophy.

Furthermore, medications that block UGCG (glucosylceramide synthase) are used to treat Fabry and Gaucher diseases [47], both of which can affect the heart [48, 49] due to glycolipid metabolism disorders. Our study indicates that blocking the UGCG–B4GalT5 axis can reduce pathological heart hypertrophy; therefore, these UGCG inhibitors may also be used to treat pressure overload-induced remodeling of the heart. However, the safety and efficacy of these inhibitors outside the context of Gaucher and Fabry diseases remain unknown, warranting further study.

## Conclusions

This study demonstrated that the inhibition of UGCG expression could partially improve remodeling of stress-induced heart hypertrophy. In addition, we revealed the interaction between B4GalT5 and UGCG, where inhibiting B4GalT5 impairs the hypertrophic effects of UGCG. Furthermore, through the regulation of mitochondrial oxidative stress and the ERK signaling pathway, these two molecules collaborate to participate in cardiac remodeling.

## Data Availability

The datasets used and/or analyzed during the current study are available from the corresponding author on reasonable request.

## Abbreviations

UGCG: UDP-glucose ceramide glycosyltransferase
B4GalT5: Beta-1,4-galactosyltransferase 5
GlcCer: Glucosylceramide
ERK: Extracellular signal-regulated kinase
NIH: National Institutes of Health
TAC: Transverse aortic constriction
AAV: Adeno-associated virus
Sh-: Small hairpin-
Col1: Collagen I
PE: Phenylephrine
β-MHC: β-Myosin heavy chain
Ad: Adenovirus
ANP: Atrial natriuretic peptide
BNP: Brain natriuretic peptide
TGF-β: Transforming growth factor-beta
LVIDd: Left ventricle internal dimension diastole
LVPWd: Left ventricle posterior wall dimension
TEM: Transmission electron microscope
MMP: Measurement of mitochondrial membrane potential
p-: Phosphorylated-
qRT–PCR: Real-time quantitative polymerase chain reaction
ROS: Reactive oxygen species
